# Natural Compound Melatonin Suppresses Breast Cancer Development by Regulating Circadian Rhythm

**DOI:** 10.3390/nu17213386

**Published:** 2025-10-28

**Authors:** Yuanli He, Chenchen Hu, Feiming Hu, Yuanjie Sun, Lin Guo, Junyi Ren, Chenying Han, Yuhui Li, Xiyang Zhang, Yubo Sun, Junqi Zhang, Sirui Cai, Yueyue Wang, Dongbo Jiang, Kun Yang, Shuya Yang

**Affiliations:** 1 Department of Immunology, Air Force Medical University, No. 169, Changle West Road, Xi’an 710032, China; yuanli5832@163.com (Y.H.); 18579121005@163.com (C.H.); 17778914060@163.com (F.H.); syjfly@163.com (Y.S.); 15389691926@163.com (L.G.); hanchenying7777777@163.com (C.H.); sunyubo000103@163.com (Y.S.); zjq000211@163.com (J.Z.); caisirui@fmmu.edu.cn (S.C.); wangyyue66@fmmu.edu.cn (Y.W.); superjames1991@foxmail.com (D.J.); 2 Yan’an Key Laboratory of Microbial Drug Innovation and Transformation, School of Basic Medicine, Yan’an University, Yan’an 716000, China; 3 School of Basic Medicine, Air Force Medical University, No. 169, Changle West Road, Xi’an 710032, China; 15993319021@163.com (J.R.); 19567685596@163.com (Y.L.); 4 Military Medical Innovation Center, Air Force Medical University, Xi’an 710032, China; zhangxiyang199272@163.com

**Keywords:** melatonin, circadian rhythm, BMAL1, ALDH3A1, glycolysis, breast cancer

## Abstract

**Background:** Breast cancer remains a major global health threat to women. While current therapies exist, their limitations necessitate novel strategies. Melatonin, an endogenous circadian regulator, has shown anti-tumor potential, but its mechanisms from a circadian perspective require further exploration. **Methods:** The anti-tumor effects of melatonin were evaluated through cell proliferation, colony formation, and apoptosis assays. Through data analysis and experimental verification at the RNA and protein levels, the regulatory effect of it on the core clock gene BMAL1 was studied. The role of BMAL1 in mediating melatonin’s suppression of glucose metabolism was assessed by measuring glucose uptake and lactate production. Downstream effector molecules of BMAL1 were identified through molecular interaction and transcriptional regulation analyses. **Results:** Melatonin significantly inhibited breast cancer cell proliferation and colony formation and induced apoptosis. Mechanistically, it upregulates the core clock gene BMAL1, which suppresses glucose metabolism. ALDH3A1 was identified as a key downstream target of BMAL1, defining a novel “melatonin-BMAL1-ALDH3A1” axis. In vivo studies confirmed that this axis effectively inhibits tumor growth without apparent toxicity, and SR8278 also shows a synergistic effect when used in combination with melatonin. **Conclusions:** Our findings elucidate the role of the “melatonin-BMAL1-ALDH3A1” axis in combating breast cancer, offering a new direction for treatment and laying the groundwork for developing precision chronotherapy-based combination regimens.

## 1. Introduction

Globally, breast cancer ranks as the most common malignant neoplasm in women, representing roughly 31% of all cancer cases diagnosed in females [1]. In 2022, more than 2.3 million new cases of breast cancer were reported worldwide, accounting for 11.6% of all new cancer cases. Based on current epidemiological trends, the global annual incidence of breast cancer is projected to exceed 3 million by 2040 [2]. The prognosis of breast cancer patients is closely related to the stage at the time of diagnosis: the 5-year survival rate of patients with localized breast cancer can reach 86%, and once metastasis occurs, the 5-year survival rate is significantly reduced to 27%. Epidemiological studies have identified various risk factors associated with the risk of breast cancer, including specific gene mutations, radiation exposure, age at menarche and unhealthy living habits [3]. At present, the clinical management strategies of breast cancer mainly include mastectomy, supplemented by radiotherapy, endocrine therapy, neoadjuvant chemotherapy, biological or targeted therapy, and the combination of these treatments [4]. Exploring adjuvant or alternative treatment strategies with new mechanisms of action is still an urgent challenge in the field of breast cancer treatment.

Melatonin (N-Acetyl-5-methoxytryptamine) is an endogenous neurotransmitter primarily synthesized by the pineal gland. Its synthesis and secretion are strictly regulated by the retinohypothalamic tract, exhibiting a robust circadian rhythm [5]. Due to its low molecular weight and amphiphilic nature, melatonin readily penetrates cellular membranes and is widely distributed throughout the body [6]. Beyond its well-established role in regulating circadian rhythms and the sleep–wake cycle, melatonin exerts potent antioxidant effects. It and its metabolites can initiate an antioxidant cascade reaction, which continuously generates free radical scavengers, thereby mitigating oxidative damage through multiple mechanisms [7]. These properties, particularly its capacity to mitigate oxidative stress, contribute significantly to its broader anti-cancer effects. Its anti-cancer mechanism of action involves the fine-tuning of a variety of cellular processes, including the induction of autophagy, modulation of mitochondrial function, triggering of endoplasmic reticulum stress, and the regulation of cell death [8]. In glioblastoma, it induces autophagy and apoptosis [9]. In renal cell carcinoma, melatonin induces a process referred to as “tumor slimming” through the enhancement of lipofuscinosis and autophagy, which is mediated by the peroxisome proliferator-activated receptor gamma coactivator 1 alpha (PGC1α) and uncoupling protein 1 (UCP1) signaling pathway [10]. In head and neck squamous cell carcinoma, melatonin influences folate metabolism through the inhibition of the MTHFD1L-formate pathway [11]. Consequently, melatonin, whether used as a monotherapy or in combination with conventional treatments, holds significant promise for advancing cancer therapy.

The biological clock is essential for the maintenance of physiological homeostasis and normal functioning of all organisms, and the core molecular mechanism consists of a transcription-translation feedback loop (TTFL) [12]. The core components include brain and muscle ARNT-Like 1 gene (BMAL1), circadian locomotor output cycles kaput (CLOCK), period (PER) and cryptochrome (CRY) [13]. Within this regulatory circuit, BMAL1 assembles with the CLOCK protein to form a heterodimer. This dimer specifically binds to the promoter regions of target genes containing E-box elements, thereby initiating and driving the transcription process of these target genes. Subsequently, PER and CRY proteins, which gradually accumulate in the cytoplasm, further form a protein complex. Upon transport into the nucleus, this complex inhibits the transcriptional activity of the BMAL1/CLOCK heterodimer through mutual interaction, thereby constituting the negative feedback regulatory component of the circuit. Upon clearance of the PER/CRY complex by intracellular degradation systems, its inhibitory effect on BMAL1/CLOCK is lifted, thereby restarting the entire circadian rhythm regulatory cycle [14]. Furthermore, BMAL1 also regulates multiple factors, including RAR-related orphan receptor A (RORA), nuclear receptor subfamily 1, group D, member 1/2 (NR1D1/2), and sirtuin 1 (SIRT1). Through its regulation of these factors, it further participates in the modulation of circadian rhythms [15].

The association between circadian rhythm disruption and cancer development is of increasing interest, and the core biological clock gene BMAL1 plays a key role in tumor suppression [16]. In hepatocellular carcinoma (HCC) and glioblastoma multiforme (GBM), abnormal expression of BMAL1 is significantly correlated with enhanced tumor cell proliferation capacity, increased invasive potential, and heightened migratory activity [17]. In pancreatic cancer, BMAL1 has been shown to regulate apoptosis and autophagy processes in cells [18]. Furthermore, in hepatocellular carcinoma, BMAL1 was shown to transcriptionally inhibit glycerol-3-phosphate acyltransferase mitochondrial (GPAM) expression in an Enhancer of zeste homolog 2 (EZH2)-dependent manner, suppressing tumor cell growth by interfering with glycolipid metabolic processes [19].

This research investigated the anti-cancer mechanisms of melatonin in breast cancer (BC). The key findings indicated that melatonin activated the BMAL1-ALDH3A1-glycolysis regulatory axis by significantly upregulating the expression of the core biological clock gene BMAL1. The activation of this pathway effectively inhibited the glycolytic process of tumor cells, disrupting their intracellular homeostasis and thus inhibiting tumor cell proliferation. The study not only reveals a new mechanism of breast cancer inhibition by melatonin through the regulation of BMAL1, a core gene of the biological clock, and its downstream metabolic pathways, but also highlights the important potential of melatonin or in combination with BMAL1 agonists in the future therapeutic strategy of breast cancer.

## 2. Materials and Methods

### 2.1. Cell Culture and Reagents

The human MCF7, SKBR3, Hela, and mouse 4T1 breast cancer cell lines (zqxzbio, Shanghai, China) were cultured in Dulbecco’s modified Eagle’s medium (DMEM) (Gibco, Waltham, MA, USA) fortified with 10% FBS and 1% antibiotics (Gibco, Waltham, MA, USA), at 37 °C in a 5% CO_2_ environment. All cell lines tested mycoplasma-negative and were authenticated using short tandem repeat (STR) profiling. Dimethyl sulfoxide (DMSO), Melatonin and SR8278 were purchased from MedChemExpress (MCE, New York, NY, USA). Melatonin and SR8278 were prepared in DMSO according to the manufacturer’s protocol, aliquoted, and stored at −80 °C.

### 2.2. Data Collection and Analysis

In this study, datasets from The Cancer Genome Atlas (TCGA) and the Gene Expression Omnibus (GEO) dataset GSE42568 were selected to conduct gene expression-related analyses. Survival analysis was performed using the “survminer” package (version 0.4.9) and “survival” package (version 3.5.7) in the R statistical environment. The Kaplan–Meier estimator was used to plot survival curves, with a *p*-value < 0.05 set as the threshold for determining statistical significance. Heatmaps of differentially expressed genes (DEGs) were constructed using the “pheatmap” package in the R programming environment. Gene Ontology (GO) enrichment analysis was implemented using the “clusterProfiler” package (version 4.10.0) combined with the “ggplot2” package (version 3.5.1) in R, covering three major categories: Molecular Function (MF), Biological Process (BP), and Cellular Component (CC). The criteria for enhanced significance were set as both *p*-value and q-value < 0.05. Additionally, Gene Set Enrichment Analysis (GSEA) was applied to detect the complete transcriptome data of all tumor samples, thereby revealing the potential mechanisms of prognosis-related genes and identifying differentially regulated pathways, with the statistical significance criterion set as a false discovery rate (FDR) < 0.05. To evaluate the binding energy and interaction mode between melatonin and BMAL1, molecular docking was performed using AutoDock Vina 1.2.2. The three-dimensional structure of melatonin was retrieved from the PubChem database, while the crystal structure of BMAL1 was obtained from the Protein Data Bank (PDB). Prior to docking, both the protein and ligand were prepared by removing water molecules, adding polar hydrogen atoms, and converting the files to PDBQT format. A grid box was set to encompass the binding domain of BMAL1, allowing sufficient space for ligand movement. The docking results were visualized using PYMOL software (version 3.0).

### 2.3. Nucleic Acid Transfection

BMAL1 siRNA and BMAL1 overexpression plasmid were purchased from TSINGKE (Beijing, China). Lipofectamine 3000 (Invitrogen, Carlsbad, CA, USA) was utilized for transfecting BMAL1 siRNA and BMAL1 plasmids. Following transfection for 48 or 72 h, it is essential to verify transfection efficiency using qRT-PCR or Western blot analysis before moving on to further experiments. All the sequences listed above can be found in Table S1.

### 2.4. Quantitative Real-Time Reverse Transcription PCR (qRT-PCR)

The processed samples underwent total RNA extraction using an RNA extraction kit (TSINGKE, Beijing, China). Subsequently, total RNA was transcribed into complementary DNA (cDNA) via one-step reverse transcription polymerase chain reaction (RT-PCR). Real-time quantitative polymerase chain reaction (qPCR) analysis was then performed using SYBR Green PCR master mix (Vazyme Biotech, Nanjing, China). Primer sequences employed in the experiment are detailed in Table S2.

### 2.5. Western Blot and Immunoprecipitation (IP) Assays

The pre-treated cells were washed once with pre-chilled phosphate-buffered saline (PBS). RIPA lysis buffer was added and placed on ice for lysis. The lysate was scraped into a centrifuge tube and centrifuged at 12,000 rpm for 15 min. The supernatant (total protein) was collected, and the cellular debris pellet was discarded. After determining total protein concentration, equal volumes of protein were loaded onto an 8% SDS-PAGE gel for electrophoresis separation. Subsequently transfer to a polyvinylidene fluoride (PVDF) membrane was performed. The membrane was blocked with 5% skimmed milk for 1–2 h, then incubated with primary antibody overnight at 4 °C. The membrane was washed with TBST, and the corresponding secondary antibody was added and incubated at room temperature for 1 h. After washing again, antibody-binding signals were captured using an ECL chemiluminescent detection kit (ZETA Life, San Francisco, CA, USA). For co-immunoprecipitation (Co-IP) experiments, cells were lysed in buffer containing protease and phosphatase inhibitors. After centrifugation to collect the supernatant, anti-BMAL1 antibody, IgG antibody, and Protein A/G PLUS agarose beads were added for overnight incubation. Following bead washing, results were analysed by Western blotting. Details of antibodies used are provided in Table S3.

### 2.6. Cell Viability and Colony Formation Assays

To evaluate the viability of the cells, a total of 5000 treated cells were plated per well in a 96-well plate, utilizing the Cell Counting Kit-8 (CCK-8) from Yisheng (Shanghai, China) to track their viability. Following a 60 min incubation period with the CCK-8 solution, the absorbance was measured at 450 nm. In the colony formation study, 400 cells were planted per 60 mm dish. Post a 2-week incubation period, cells were immobilized with paraformaldehyde for a duration of 10 min. Following fixation, the cells were stained with crystal violet (Solarbio, Beijing, China), and the colonies were counted using ImageJ software (version 1.54p).

### 2.7. 5-Ethynyl-2′-Deoxyuridine (EdU) Incorporation Assay

The 5-Ethynyl-2′-deoxyuridine (EdU) experiment was conducted using the EdU Assay Kit (Beyotime Biotechnology, Shanghai, China). The specific procedure was as follows: cells were first incubated with the EdU solution for 4 h, followed by brief fixation with a 4% paraformaldehyde solution. To enhance cell permeability, cells were treated with PBS containing 0.3% Triton X-100. Next, cells were placed in Click reagent solution for 30 min at room temperature for staining. Nuclear counterstaining was performed using DAPI (Beyotime Biotechnology, Shanghai, China). Finally, images were acquired using a Thermo Fisher Scientific EVOS M5000 fluorescence microscope (Thermo Fisher Scientific, Waltham, MA, USA).

### 2.8. Chromatin Immunoprecipitation (ChIP)-qPCR

Chromatin immunoprecipitation (ChIP) experiments were conducted using the SimpleChIP Plus Enzymatic Chromatin IP Kit (Cell Signaling Technology, Danvers, MA, USA), with procedures strictly adhering to the protocol. The procedure was as follows: approximately 1.2 × 10^7^ MCF7 cells were taken and fixed using a 1% formaldehyde solution; glycine was subsequently added to terminate the cross-linking reaction. Thereafter, the adherent cells were scraped, and the cell pellet was collected by centrifuging at 2000× *g* for 5 min. The DNA was digested to an appropriate length using micrococcal nuclease, and the enzymatic reaction was terminated by adding 0.5 M EDTA. Following ultrasonic disruption of the nuclear membrane, the supernatant containing cross-linked chromatin was collected. BMAL1 antibody and IgG antibody were added to the samples and incubated overnight at 4 °C. Subsequently, magnetic beads were added to each reaction system, and incubation was continued under continuous agitation. Following incubation, the magnetic beads were sequentially washed using buffers. Finally, chromatin was eluted from the magnetic beads, and reverse cross-linking was performed. The samples were purified via spin columns and detected using real-time quantitative PCR (qRT-PCR). Primer sequences for ChIP-qPCR are provided in Table S2.

### 2.9. Immunohistochemical Staining

Paraffin-embedded tumor tissue sections underwent dewaxing and rehydration, followed by antigen retrieval. Sections were blocked with 3% bovine serum albumin (BSA) and then incubated overnight at 4 °C with the following primary antibodies: Ki67 (1:300, Abcam, Cambridge, UK), BMAL1 (1:200, Proteintech, Rosemont, IL, USA), and ALDH3A1 (1:200, Origene, Rockville, MD, USA). Sections were washed with phosphate-buffered saline (PBS), followed by staining with 3,3′-Diaminobenzidine (DAB) peroxidase substrate (Vector Laboratories, Oxfordshire, UK). Finally, stained sections were digitally acquired and processed using a digital slide scanning system (WINMEDIC, Shandong, China).

### 2.10. Sphere-Formation Assay

A volume of 150 μL of 1.5% agarose solution was dispensed into each well of a 48-well culture plate. After the agarose had solidified, 10,000 tumor cells were suspended in 200 μL of culture medium, and this suspension was applied onto the agarose-coated surface. Subsequently, the plate was centrifuged at 1000× *g* for 10 min, followed by incubation under standard culture conditions.

### 2.11. Flow Cytometry

Apoptosis assays employed Annexin V-FITC/PI dual staining (BestBio, Shanghai, China). Following staining, samples were analysed by flow cytometry to distinguish viable cells, apoptotic cells, and necrotic cells. Data acquisition and subsequent analysis were performed using Novo Express software (version 1.4.1) (ACEA Biosciences, San Diego, CA, USA).

### 2.12. Metabolic Assay

Metabolic assays for glucose uptake and lactate production in the culture medium of breast cancer cells were conducted using commercially available kits (Beyotime Biotechnology, Shanghai, China).

### 2.13. Dual Luciferase Reporter Gene Assay

The interaction between BMAL1 and the ALDH3A1 promoter was validated using a dual luciferase reporter assay. The ALDH3A1 promoter fragment (region 841–1141 relative to the transcription start site) was cloned into the pGL3-Basic vector (GeneCreate Biotech, Wuhan, China). Twenty-four hours later, the reporter plasmid, pRL-TK control vector, and BMAL1 overexpression plasmid (or empty vector) were co-transfected into cells using Lipofectamine 3000. Detection was performed using a dual luciferase reporter assay kit (Vazyme Biotech, Nanjing, China).

### 2.14. Animal Experiment

Seven-week-old female Balb/c mice were maintained under a 12 h light/12 h dark cycle. Under isoflurane anesthesia, mice received injections of 5 × 10^5^ 4T1 cells into the fat pad of the fourth mammary gland. Ten days post-injection, the mice were randomly assigned to four experimental groups using a computer-generated random number table (n = 5 per group) and administered intraperitoneally with either phosphate-buffered saline (PBS), melatonin (40 mg/kg), SR8278 (20 mg/kg), or a combination of SR8278 and melatonin, twice weekly for a duration of three weeks. To maintain the circadian rhythm of mice, drug administration was conducted at night throughout the whole experiment. Images were obtained utilizing a PerkinElmer IVIS preclinical in vivo imaging system. At the study endpoint, all mice were euthanized by CO_2_ asphyxiation. Tumors and relevant organs were then collected and processed with care to preserve structural integrity. The samples were gently rinsed with PBS, weighed on a precision balance, and fixed in 4% paraformaldehyde for subsequent examination. The animal study was reviewed and approved by the Ethics Committee of the Fourth Military Medical University (approval number: 20230930).

### 2.15. Statistical Analysis

Statistical analyses and graphical representations were performed using GraphPad Prism version 8.0.2. Comparisons between two groups were conducted using Student’s *t*-test, while one-way analysis of variance (ANOVA) was employed for comparisons involving multiple groups. Each experiment was independently replicated a minimum of three times, and the results are expressed as means ± standard deviation (SD). Statistical significance is denoted as follows: * *p* < 0.05, ** *p* < 0.01, *** *p* < 0.001, and **** *p* < 0.0001. NS indicates no significant difference.

## 3. Results

### 3.1. Melatonin Inhibits Cell Proliferation

**Figure 1:**
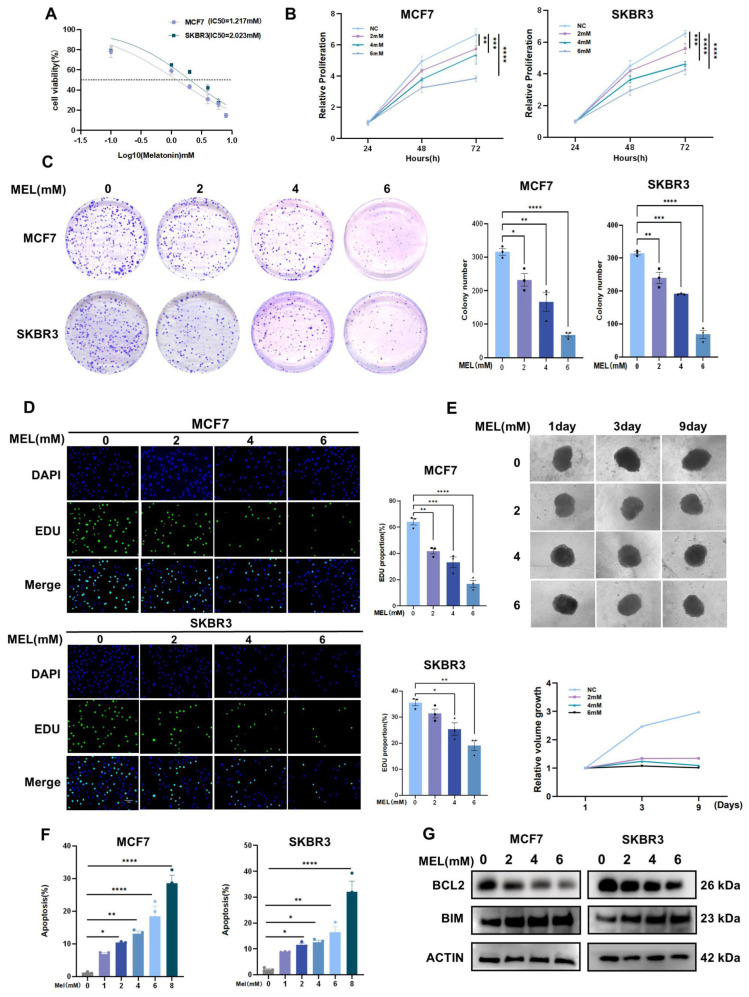
Melatonin inhibits cell proliferation. (**A**) MCF7 and SKBR3 cell lines were exposed to a series of melatonin concentrations (0, 1, 2, 4, 6, and 8 mM) for a duration of 24 h. Subsequently, cell viability was assessed utilizing the CCK8 assay. The half-maximal inhibitory concentration (IC_50_) values were determined through analysis with GraphPad Prism software. (**B**) The viability of MCF7 and SKBR3 cells exposed to varying concentrations of melatonin (0, 2, 4, and 6 mM) for 72 h was assessed at designated time points using the CCK8 assay. (**C**) The proliferative potential of MCF7 and SKBR3 cells exposed to varying concentrations of melatonin (0, 2, 4, and 6 mM) was evaluated through a colony formation assay. (**D**) The proliferative capacity of cells under various treatment conditions was evaluated using EdU (5-ethynyl-2′-deoxyuridine) incorporation assays. (**E**) Micrograph of multicellular tumor spheroid generated from SKBR3 after treatment with 0, 2, 4, or 6 mM melatonin. (**F**) Statistical analysis of apoptotic cells of MCF7 cells and SKBR3 cells under 24 h melatonin treatment (n = 3). (**G**) Western blot analysis of apoptosis-associated proteins in BC cells following 24 h of melatonin treatment. * *p*  <  0.05, ** *p*  <  0.01, *** *p*  <  0.001, **** *p* < 0.0001.

Given melatonin’s demonstrated potential anti-tumor activity, to further elucidate its role and effect characteristics in breast cancer, two breast cancer cell lines—MCF7 and SKBR3—were exposed to a gradient concentration of melatonin and incubated under appropriate culture conditions for 24 h. Subsequently, cell viability was quantified across different treatment groups using the Cell Counting Kit-8 (CCK-8) assay, which measures absorbance values. Results demonstrated a gradient decrease in cell viability with increasing melatonin concentrations. The half-maximal inhibitory concentration (IC_50_) for MCF7 cells was 1.217 mM, while that for SKBR3 cells was 2.023 mM (Figure 1A). Based on these IC50 values, four different concentrations of melatonin were selected for subsequent experiments. Melatonin reduced the viability of BC cells (Figure 1B), as well as their colony-forming (Figure 1C) and proliferative (Figure 1D) abilities. Tumor cells are cultured in conditions lacking suitable attachment surfaces, thereby promoting cellular aggregation into spheroids. This tumor spheroid model simulates the three-dimensional growth microenvironment of in vivo tumors, enabling subsequent evaluation of biological characteristics such as tumor cell self-renewal, drug resistance, and invasive capacity [20]. SKBR3 cells were cultured to form dense spheroids. After treatment with melatonin, their spheroid-forming capacity and size were measured, and the results showed that melatonin could inhibit the growth of tumor spheroids (Figure 1E). Notably, melatonin suppresses cell proliferation in a dose-dependent manner. Exposure to 2 mM melatonin significantly inhibited cell proliferation; furthermore, elevating the concentration to 4 and 6 mM produced a more substantial inhibitory effect in both MCF7 and SKBR3 cell lines. The findings from the apoptosis assays further indicated that the cytotoxic effects of melatonin exhibit a dose-dependent relationship (Figure 1F). Western blot analysis demonstrated that melatonin treatment significantly increased the expression of Bim, a pro-apoptotic protein, while concurrently reducing the levels of Bcl-2, an anti-apoptotic protein (Figure 1G). In summary, our findings indicate that melatonin administration demonstrated a significant and dose-dependent inhibitory impact on cell proliferation.

### 3.2. Melatonin Inhibits Tumor Cell Growth by Regulating BMAL1

**Figure 2:**
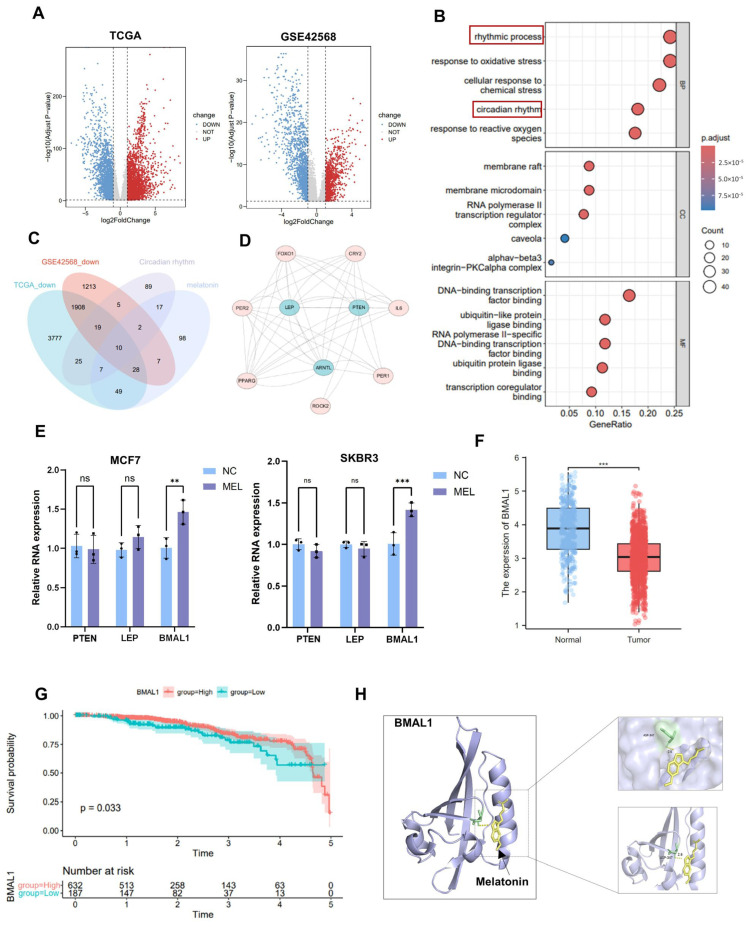
Melatonin inhibits tumor cell growth by regulating BMAL1. (**A**) Volcano plots represent DEGs in each comparison combination. (**B**) GO analysis. (**C**) Venn diagram of overlapping genes. (**D**) Feature gene PPI network diagram. (**E**) Gene expression levels of PTEN, LEP, and BMAL1 in melatonin and ctrl. (**F**) Expression level of BMAL1 in normal breast tissues versus BC tissues in the GSE42568 dataset. (**G**) Survival analysis of BC patients with different BMAL1 levels. (**H**) Molecular docking patterns of melatonin and BMAL1. ns, not significant, ** *p*  <  0.01, *** *p*  <  0.001.

In this study, a differential analysis was conducted on two microarray datasets (TCGA and GSE42568), and subsequently, a volcano plot was generated to visually present the differentially expressed genes (Figure 2A). Gene Ontology (GO) enrichment analysis of differentially expressed genes (DEGs) identified significant associations with biological processes (BP), cellular components (CC), and molecular functions (MF). The top 5 GO items were listed (Figure 2B). The results revealed that the rhythmic process, the circadian rhythm, was significantly altered. Notably, the enrichment of circadian rhythm-related processes suggests that dysregulation of circadian genes may contribute to BC proliferation. An intersection analysis was conducted on the results derived from the four datasets, yielding ten common key feature genes (Figure 2C). These ten identified genes were subsequently submitted to the STRING database to construct a protein–protein interaction (PPI) network (Figure 2D). We observed that three genes were situated within the network’s core region and exhibited a higher number of connecting edges, suggesting they may perform key regulatory functions within the melatonin action mechanism. To further elucidate the expression patterns of these key genes, RNA was extracted from BC cells treated with 2 mM melatonin for 24 h, followed by quantitative real-time reverse transcription PCR analysis. Results revealed a significant upregulation trend in BMAL1 gene expression compared to control cells (Figure 2E). Then, we explored the expression status of BMAL1 in BC samples and found that BMAL1 was significantly down-regulated in BC tissues compared with normal breast tissues (Figure 2F). The survival analysis indicated that patients exhibiting reduced BMAL1 expression levels are associated with decreased overall survival durations (Figure 2G). Molecular docking analysis revealed that BMAL1 and melatonin could form a stable binding conformation (Figure 2H), with a binding energy of −5.193 kcal/mol. These results collectively suggest the possibility of direct binding between the two, but this conclusion requires further experimental verification. Data analysis and experimental verification show that BMAL1, as a key circadian rhythm-related gene, is down-regulated in breast cancer and is associated with poor prognosis. BMAL1 may be an important mediating mechanism of melatonin regulating breast cancer.

### 3.3. Melatonin Regulates BMAL1 to Affect Glycolysis via ALDH3A1 in BC Cells

**Figure 3:**
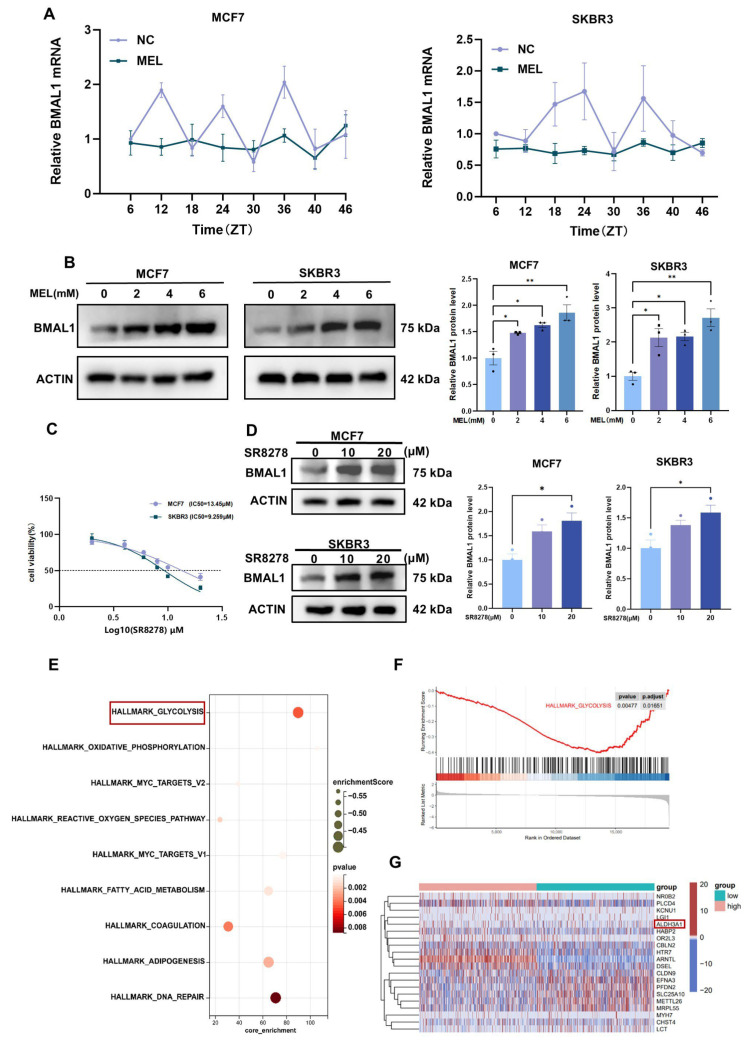
Melatonin regulates BMAL1 to affect glycolysis via ALDH3A1 in BC cells. (**A**) mRNA expression of BMAL1 in MCF7 and SKBR3 cells at the indicated times. Each point represents mean  ±  SEM values of three independent assays (n  =  3). (**B**) Western blot assay of BMAL1 protein alteration after 24 h melatonin (0, 2, 4, 6 mM) treatment in BC cells. (**C**) IC_50_ value of 24 h SR8278 treatment on BC cells. (**D**) Western blot assay of BMAL1 protein alteration after 24 h SR8278 (0, 10, 20 μM) treatment in BC cells. (**E**,**F**) This dot plot visualizes gene set enrichment analysis (GSEA) results for hallmark gene sets. (**G**) Heat map of DEGs. Differentially expressed genes (DEGs) were identified in the integrated expression matrix by selecting *p* < 0.05 and |log fold change (FC)| > 1.0 as thresholds. * *p*  <  0.05, ** *p*  <  0.01.

Melatonin treatment reprogrammed the circadian expression profile of BMAL1 mRNA, resulting in a transition from a high-amplitude rhythm to a low-amplitude oscillation (Figure 3A). Melatonin generates a robust and stable BMAL1 expression landscape. Western blot analysis revealed that melatonin enhances BMAL1 protein expression in a manner dependent on its concentration (Figure 3B). SR8278 is a synthetic competitive antagonist of the nuclear receptor REV-ERB. It exerts its effect by competitively binding to target DNA sequences of REV-ERB, such as the REV-ERB response element (RORE) within the BMAL1 promoter region. This action releases the inhibitory control of REV-ERB over BMAL1 transcription, thereby promoting the transcriptional expression of BMAL1 [21]. Using the CCK-8 assay to detect and calculate the half-maximal inhibitory concentration (IC_50_), it was found that SR8278 exhibited a pronounced dose-dependent inhibitory effect on the viability of both MCF7 and SKBR3 cell lines, with successful fitting yielding IC_50_ values for both cell types (Figure 3C). Further Western blot analysis revealed that following treatment with SR8278, BMAL1 protein expression levels were significantly upregulated in both cell lines. This finding correlates with the inhibitory effect of SR8278 on cell viability (Figure 3D). SR8278 and the endogenous hormone melatonin converge on elevating BMAL1 protein levels. These preliminary research results indicate that melatonin can effectively promote the upregulation of BMAL1 protein expression levels. However, the specific molecular mechanism of its action still needs to be systematically clarified through subsequent experiments. This suggests its role as an intrinsic molecular regulator. Notably, further analysis of BMAL1-regulated differentially expressed genes (DEGs) revealed significant enrichment in glycolytic metabolic pathways. Glycolysis not only supplies ample ATP and intermediate metabolites for rapid tumor proliferation but also plays an irreplaceable role in critical progression processes such as tumor invasion, metastasis, and drug resistance development. This suggests BMAL1 may influence the biological behaviour of breast cancer by regulating the glycolytic pathway (Figure 3E) and exhibits a negative correlation with glycolysis (Figure 3F). Heatmap plot of the top genes with the biggest variances after BMAL1 downregulation (Figure 3G). We found ALDH3A1 as one of the differentially expressed genes. Aldehyde dehydrogenase 3A1 (ALDH3A1) is an enzyme that relies on NAD^+^ as a cofactor to catalyze the oxidation of a range of endogenous and exogenous aldehydes into their corresponding carboxylic acids [22]. Some studies have shown that ALDH3A1 promotes glycolysis and lactate accumulation through activation of the LDHA pathway; inhibition of ALDH3A1 reduces lactate levels and inhibits tumor cell proliferation [23]. Collectively, our data support a model whereby melatonin upregulates the expression of BMAL1, which is associated with ALDH3A1, thereby affecting the glycolytic process and ultimately influencing the proliferation of breast cancer cells.

### 3.4. BMAL1 Inhibits Tumor Growth with Melatonin Potentiating the Effect in BC Cells

**Figure 4:**
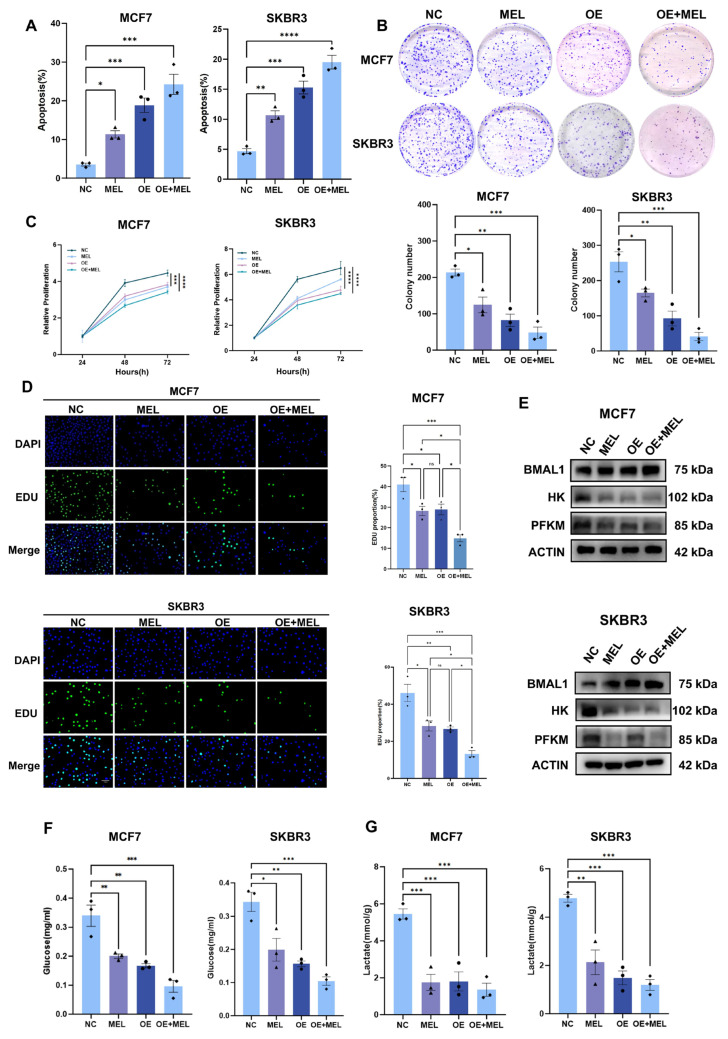
BMAL1 inhibits tumor growth with melatonin potentiating the effect in BC cells. (**A**) Apoptosis assay. (**B**) Colony formation. (**C**) CCK8 assay. (**D**) EdU assay. (**E**) MCF7 and SKBR3 cells were engineered to overexpress BMAL1, followed by the assessment of protein expression levels using Western blot analysis. (**F**) Glucose uptake. (**G**) Lactate production. ns, not significant, * *p*  <  0.05, ** *p*  <  0.01, *** *p*  <  0.001, **** *p* < 0.0001.

Given that BMAL1 may represent a downstream target of melatonin, this study further investigated the regulatory effects of BMAL1 overexpression on the biological behaviour of breast cancer cells. Following construction of a BMAL1 overexpression plasmid and its transfection into BC cells, apoptosis levels were assessed via flow cytometry. Results demonstrated a significantly elevated apoptosis rate in the BMAL1 overexpression group compared to the empty vector control group (Figure 4A). Colony formation assessment, CCK-8, and EdU assays showed a significantly lower clonogenicity, cell viability, and proportions of proliferating cells in MCF7 and SKBR3 cells with BMAL1 overexpression (Figure 4B–D). Additionally, expression of glycolysis-related genes was decreased at the protein level, and the decrease was more pronounced when melatonin was co-administered (Figure 4E). Prior research indicates that BMAL1 may modulate the proliferative potential of breast cancer cells through the regulation of glycolytic processes. To investigate the involvement of glycolytic dysregulation in breast cancer progression, this study evaluated two critical metabolites within the glycolytic pathway: glucose uptake and lactate production. The findings revealed that, consistent with expectations, overexpression of BMAL1 markedly decreased both the glucose uptake capacity (Figure 4F) and lactate production (Figure 4G) in breast cancer cells. In summary, overexpression of BMAL1 exerts an inhibitory effect on breast cancer cells by promoting apoptosis, suppressing proliferation, and downregulating glycolysis-related pathways. The inhibitory effects of BMAL1 overexpression were potentiated by co-treatment with melatonin, indicating that melatonin’s anti-tumor effects are consistent with and may amplify the BMAL1-mediated suppression of glycolysis and proliferation.

### 3.5. Silencing of BMAL1 Partially Rescues Melatonin-Mediated Attenuation of BC Cell Proliferation and Glycolysis

**Figure 5:**
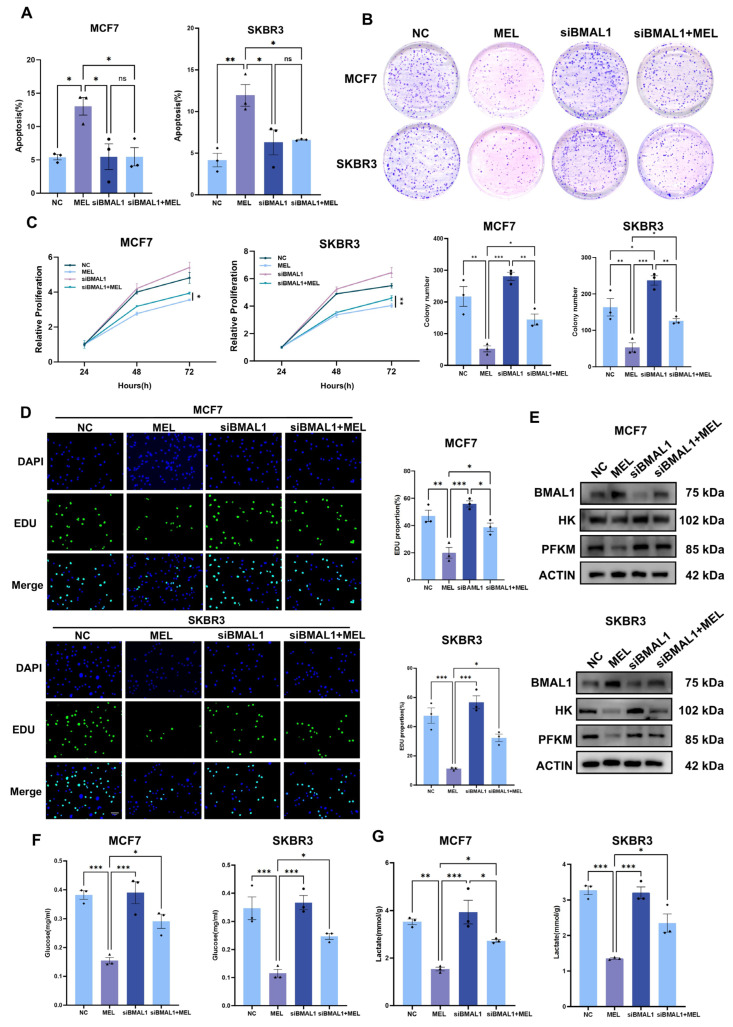
Silencing of BMAL1 partially rescues melatonin-mediated attenuation of BC cell proliferation and aerobic glycolysis. (**A**) Apoptosis assay. (**B**) Colony formation. (**C**) CCK8 assay. (**D**) EDU assay. (**E**) MCF7 and SKBR3 cells with BMAL1 Silencing, after which the protein levels were determined by Western blot. (**F**) Glucose uptake. (**G**) Lactate production. ns, not significant, * *p*  <  0.05, ** *p*  <  0.01, *** *p*  <  0.001.

To investigate whether silencing BMAL1 can reverse the decrease in breast cancer cell viability and clonogenicity induced by melatonin treatment in vitro, we conducted relevant experiments. The results showed that silencing BMAL1 reduced cell apoptosis (Figure 5A); further observations via colony formation assays and CCK-8 assays (Figure 5B,C) revealed that it could restore the proliferative capacity of breast cancer cells (Figure 5D). Meanwhile, the protein expression levels of glycolysis-related genes were restored (Figure 5E), and both glucose uptake (Figure 5F) and lactate production (Figure 5G) were also recovered. These results indicate that silencing BMAL1 can reverse the pro-apoptotic effect of melatonin treatment on breast cancer cells, restore their proliferative activity and glycolytic function, and thus validate from the reverse perspective that BMAL1 plays a key mediating role in the regulation of breast cancer by melatonin.

### 3.6. BMAL1 Transcriptionally Inhibits ALDH3A1 Expression in BC Cells

**Figure 6:**
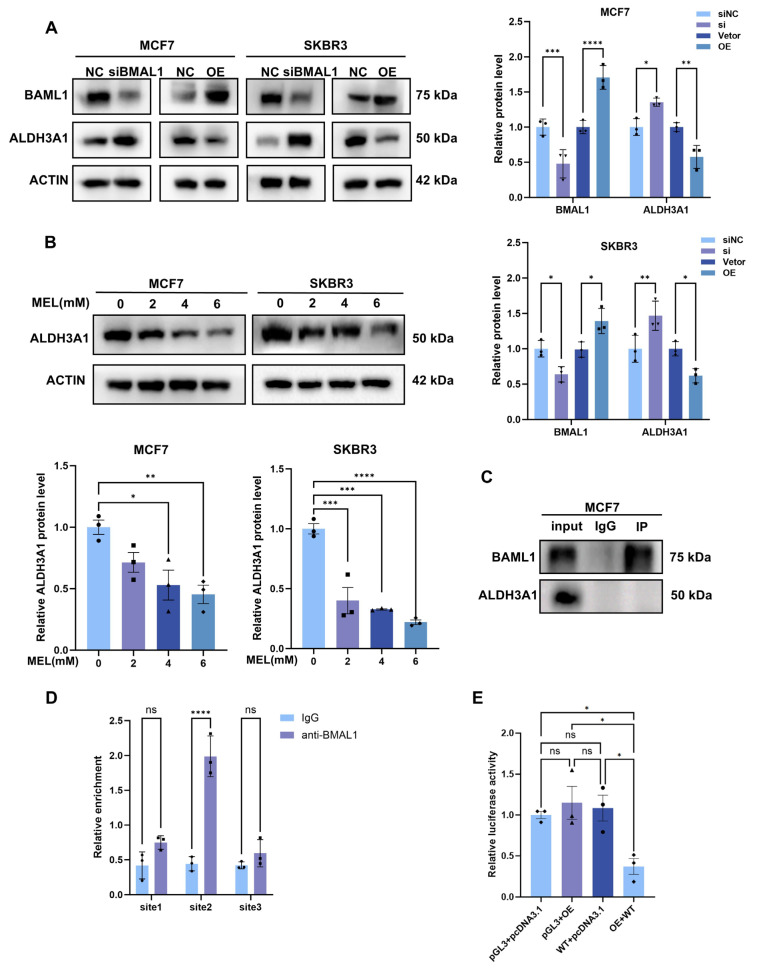
BMAL1 transcriptionally inhibits ALDH3A1 expression in BC cells. (**A**) Western blot analyses were conducted to evaluate the expression levels of BMAL1 and ALDH3A1 in MCF7 and SKBR3 cell lines following designated treatments (**B**) Western blot results of ALDH3A1 protein alteration with 24 h melatonin treatment. (**C**) To detect the interaction between the specified protein and BMAL1, lysates from breast cancer cells expressing this protein were subjected to immunoprecipitation using anti-BMAL1 antibody and IgG isotype control antibody. Following processing, the immunoprecipitates were analysed by Western blot using the designated antibody. (**D**) Using ChIP-qPCR technology, the enrichment levels of potential BMAL1 binding sites on the ALDH3A1 promoter were detected. (**E**) Relative luciferase activity driven by the ALDH3A1 promoter was measured in Hela cell lines following designated treatments. ns, not significant, * *p*  <  0.05, ** *p*  <  0.01, *** *p*  <  0.001, **** *p* < 0.0001.

Drawing upon the findings from prior experiments, we hypothesized that ALDH3A1 functions as a downstream gene regulated by BMAL1 in BC cells. Western blot analysis revealed that in both MCF7 and SKBR3 breast cancer cell lines, the protein expression levels of ALDH3A1 exhibited a negative correlation with BMAL1 expression status. Upon silencing BMAL1, ALDH3A1 expression was significantly upregulated in the cells; conversely, when BMAL1 was overexpressed via transfection with an overexpression plasmid, ALDH3A1 expression was markedly reduced (Figure 6A). Western blot analysis indicates that melatonin treatment resulted in decreased ALDH3A1 protein expression levels. Considering this finding alongside the regulatory relationship between BMAL1 and ALDH3A1, this downregulation effect may be associated with melatonin’s upregulation of BMAL1 expression (Figure 6B). To investigate how BMAL1 influences ALDH3A1, co-immunoprecipitation (co-IP) assay was performed. The results showed that BMAL1 did not directly interact with ALDH3A1 in MCF7 cells (Figure 6C). BMAL1 is a transcription factor that has been extensively studied to date. Its core mechanism of action involves binding to the promoter regions of downstream target genes, thereby initiating and activating the transcription process of these target genes. Chromatin immunoprecipitation (ChIP) analysis demonstrated that BMAL1 is capable of binding to site 2 within the promoter region of ALDH3A1 (Figure 6D). Luciferase reporter assay unveiled that the site2 was critical to BMAL1-mediated suppression of ALDH3A1 expression (Figure 6E). These findings thus demonstrate that BMAL1 can specifically bind to site 2 of the ALDH3A1 promoter, thereby suppressing ALDH3A1 expression at the transcriptional level. This establishes ALDH3A1 as a downstream target gene regulated by BMAL1.

### 3.7. Melatonin and SR8278 Collaboratively Suppress Tumor Growth In Vivo

**Figure 7:**
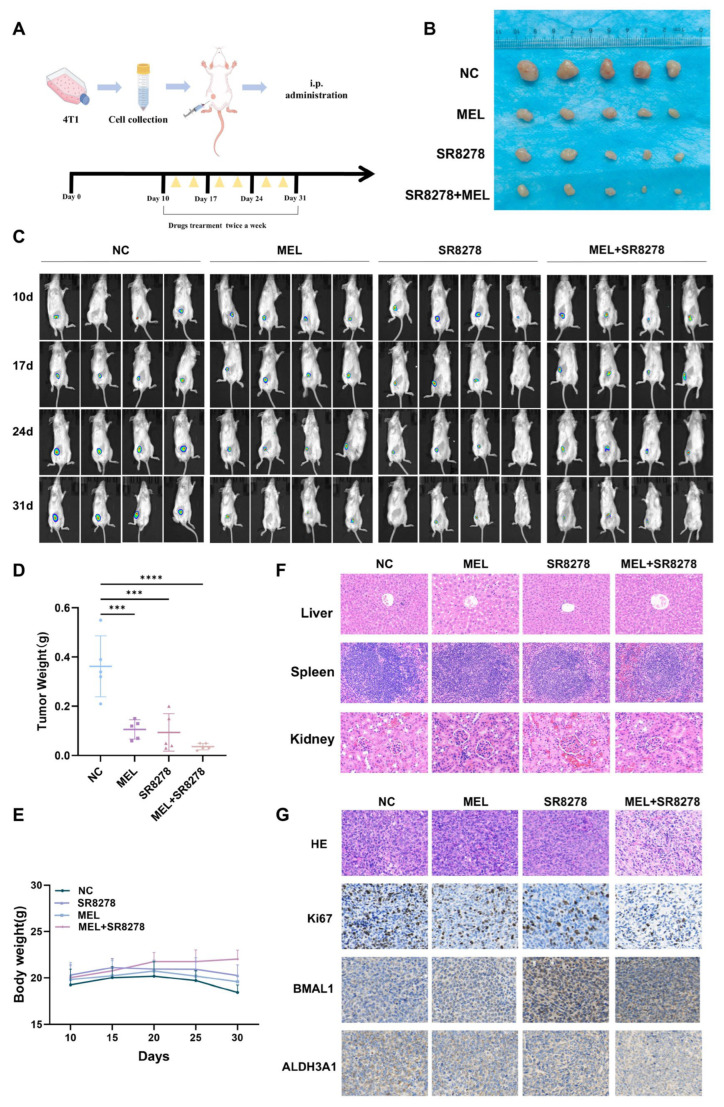
Melatonin and SR8278 collaboratively suppress tumor growth in vivo. (**A**) Flowchart Illustrating the Development of In Vivo Models and the Administration of Pharmacological Treatments. (**B**) Following a three-week treatment period with melatonin, SR8278, or a combination of both, 4T1 tumors were isolated from Balb/c mice, and the variations in tumor size across the different treatment groups were assessed and compared. (**C**) During the treatment cycle, in vivo imaging was performed at various time points on 4T1 tumor-bearing Balb/c mice. By dynamically acquiring imaging data, the tumor growth status of mice in different treatment groups was observed and monitored. (**D**) Comparison of tumor weights among different treatment groups in 4T1 tumor-bearing Balb/c mice. (**E**) Changes in body weight of Balb/c mice bearing 4T1 tumor during treatment. (**F**) H&E staining results of major organs in 4T1 tumor-bearing Balb/c mice. (**G**) Representative images depicting the expression levels of Ki67, BMAL1, and ALDH3A1 proteins in tumor tissues obtained from Balb/c mice subjected to treatment with melatonin, SR8278, or a combination of both. Scale bar = 150 µm. *** *p*  <  0.001, **** *p* < 0.0001.

Subsequently, animal models were established to examine the effects of melatonin and SR8278 on the tumorigenesis of 4T1 cells in vivo (Figure 7A). The findings demonstrated that treatment with either melatonin or SR8278 significantly inhibited tumor growth, with their combined administration resulting in a further reduction in tumor size (Figure 7B–D). No significant differences in body weight were observed among the experimental groups (Figure 7E). Consistent with expectations, assessments revealed no evidence of nephrotoxicity, hepatotoxicity, or splenic toxicity across all four groups, suggesting that melatonin and SR8278 do not induce severe toxicity or adverse effects on these organs (Figure 7F). Furthermore, immunohistochemical analysis indicated that the combined treatment with SR8278 and melatonin markedly upregulated BMAL1 expression while concurrently suppressing the expression of Ki67 and ALDH3A1 (Figure 7G).

## 4. Discussion

The development and progression of tumors, such as endometrial, prostate, lung, colon, hepatocellular and ovarian cancers, have been shown to be closely related to disruption of circadian rhythms [24]. Chronic circadian disruption disrupts key physiological processes such as cell proliferation, inflammatory response, DNA repair and metabolism, thereby increasing an individual’s risk of developing cancer [25]. Of the many types of cancer, the association between circadian rhythm disorders and breast cancer is of particular interest, with correlations dating as far back as the 1960s [26]. Studies have shown that circadian rhythm disruption is an important risk factor for breast cancer development. At the same time, the core circadian genes themselves may have tumor suppressor functions [27]. Together, this reveals that the mechanisms that regulate circadian rhythms not only help to elucidate the pathogenesis of breast cancer, but may also be a potentially effective therapeutic strategy. A deeper understanding of the molecular links between circadian rhythms and breast cancer will provide a key scientific basis for optimising existing therapies and opening up new avenues of treatment.

As a core circadian rhythm-regulating hormone and a potent antioxidant, melatonin plays a complex pleiotropic role in breast cancer development. Its well-documented capacity to mitigate oxidative stress and regulate redox homeostasis underpins its broad anti-tumor effects, providing an important molecular basis for the therapeutic strategies described above [28]. Beyond its direct antioxidant effects, melatonin’s regulatory functions extend to multiple cancer-related pathways, particularly demonstrating inhibition of hormone signalling and malignant tumor phenotypes in breast cancer. Research confirms that melatonin effectively antagonises oestrogen-mediated malignant biological behaviours in breast cancer cells. At the molecular level, this antagonistic effect operates through two mechanisms: firstly, by enhancing sensitivity to oestrogen receptor modulators (such as tamoxifen), thereby aiding these drugs in more efficiently blocking the oestrogen receptor signalling pathway [29]; secondly, by significantly downregulating the expression levels of oestrogen-regulated proteins, proto-oncogenes, and growth-promoting factors, inhibiting oestrogen-dependent tumor initiation and progression at multiple points along the signalling cascade from upstream to downstream [30]. Melatonin demonstrates an inhibitory effect on aromatase activity through the modulation of cyclooxygenase gene expression in individuals with postmenopausal breast cancer [31]. Notably, the anti-tumor activity of melatonin is not limited to hormone receptor-positive subtypes, but it also plays an important role in triple-negative breast cancer [32]. Clinical observations have shown that local radiofrequency ablation combined with melatonin therapy can significantly improve the clinical outcomes of patients with early-stage lung cancer and multiple pulmonary nodules. Specifically, it can maximize the protection of lung function structure and effectively inhibit the malignant progression of nodules in non-ablated areas [33]. Moreover, the study has also confirmed that melatonin, as a safe auxiliary intervention measure, has clear effects in improving patients’ sleep and quality of life [34]. These findings not only establish the auxiliary value of melatonin in comprehensive treatment, but also lay a solid foundation for subsequent clinical dose exploration and application. In this study, we further demonstrated through a series of experiments that melatonin treatment significantly inhibits the proliferative activity of breast cancer cells while effectively inducing apoptosis, thereby confirming its antitumor effects on breast cancer cells. To elucidate the molecular mechanisms underlying melatonin’s action and investigate its potential association with circadian rhythm regulation pathways, we detected at the protein level that melatonin treatment specifically upregulates the expression of the core circadian transcription factor BMAL1. Subsequent molecular docking analysis revealed a potential binding site and interaction possibility between melatonin and the BMAL1 protein. This prediction suggests a possible direct molecular interaction between the two, although the binding site requires further validation through subsequent experiments. Although molecular docking analysis suggests a potential direct interaction between melatonin and BMAL1, the upregulation of BMAL1 by melatonin may also involve indirect mechanisms. The central circadian clock operates through a complex transcription–translation feedback loop, and melatonin can modulate this system by activating membrane receptors such as MT1 and MT2, thereby triggering intracellular signaling pathways that ultimately influence BMAL1 transcription. For instance, studies have demonstrated that melatonin ameliorates hepatic fibrosis via melatonin receptor 2 (MT2)-mediated upregulation of BMAL1 and antioxidative enzymes [35]. In a separate study on circadian disruption-induced endochondral ossification defects, melatonin receptor 1 (MT1) was shown to periodically activate AMPKβ1 phosphorylation, which destabilizes the core clock protein CRY1 and promotes BMAL1 expression, thereby coordinating rhythms in cell proliferation and matrix synthesis [36]. Future studies should employ experimental approaches to elucidate whether the observed regulation occurs through direct binding or indirect mechanisms.

Functionally, melatonin treatment effectively inhibited the glycolytic metabolism of breast cancer cells. Critically, through functional validation, we have identified for the first time that BMAL1 is a key downstream effector molecule mediating melatonin-induced reprogramming of breast cancer cell glucose metabolism (manifested as glycolytic inhibition) and consequently inhibiting tumor cell growth. This finding provides important mechanistic insights into the anti-cancer function of melatonin through core circadian components. At the same time the circadian mechanism has an independent negative feedback loop for which antagonist or agonist-like pharmacological agents have been developed. Among these compounds, SR8278, a synthetic competitive antagonist of the nuclear receptor REV-ERB, relieves REV-ERB-mediated transcriptional repression of BMAL1 by directly binding to the REV-ERB response element (RORE) within the BMAL1 promoter region [37]. SR8278 represents a promising therapeutic candidate for Duchenne muscular dystrophy, attributable to its favorable safety profile and protective effects. The present study confirmed its effectiveness in suppressing tumor growth and distant metastasis in a mouse homozygous tumor model, and a significant synergistic anti-tumor effect was observed when SR8278 was combined with melatonin.

This study further identifies ALDH3A1 as a key downstream effector molecule of BMAL1 and a potential target for cancer therapy. ALDH3A1 is involved in metabolic regulation by catalysing the oxidation of multiple endogenous and exogenous aldehydes [38]. A substantial body of evidence indicates that the aberrant overexpression of ALDH3A1 is significantly correlated with the progression of malignant tumors [39]. Bioinformatics analyses also suggested that ALDH3A1-mediated perturbation of the glycolysis metabolic pathway was significantly associated with p53 mutation status and poor prognosis in lung adenocarcinoma [40]. Importantly, we confirmed through mechanistic studies that ALDH3A1 is a direct transcriptional target gene of BMAL1, thus establishing a new molecular bridge between core circadian regulation and reprogramming of tumor glucose metabolism.

One limitation of this study lies in the fact that the mechanism exploration was mainly based on two cell line models, MCF7 and SKBR3. Although these models provided crucial evidence for revealing the ‘melatonin-BMAL1-ALDH3A1’ regulatory axis, the applicability of this mechanism in triple-negative breast cancer (TNBC) still needs further verification. It is worth noting that some literature indicates that melatonin itself also exhibits anti-tumor activity in TNBC models (such as MDA-MB-231 cells), including inhibiting cell viability and enhancing the sensitivity to chemotherapy drugs. Additionally, previous data analysis showed that the core molecule BMAL1 is expressed in various breast cancer cell lines, including TNBC [41,42]. The wide applicability and therapeutic potential of this discovery still need to be further verified in various subtypes of breast cancer. In conclusion, our findings reveal a novel melatonin-BMAL1-ALDH3A1 regulatory axis in breast cancer. Mechanistically, melatonin upregulates the core clock protein BMAL1, which transcriptionally represses its downstream target ALDH3A1, thereby effectively blocking the glycolytic program in tumor cells and suppressing cancer progression. Furthermore, pharmacological activation of BMAL1 using the agonist SR8278 recapitulates this anti-tumor effect and demonstrates significant synergy with melatonin. The aforementioned findings of this study provide profound insights into the molecular mechanisms underlying melatonin’s anti-cancer effects, laying a theoretical foundation for subsequent tumor intervention research targeting this pathway.

## 5. Conclusions

This study not only identifies a novel melatonin-driven signaling pathway dependent on the core circadian gene BMAL1 but, more importantly, integrates circadian regulation, metabolic reprogramming, and tumor suppression into a coherent framework. Targeting this pathway (e.g., via combination strategies employing SR8278 and melatonin) represents a highly promising new direction for future breast cancer treatment research. Our findings may provide a basis for developing innovative chronotherapy-based combination treatments, and the mechanistic insights revealed herein merit further exploration in future translational studies.

## Data Availability

The original contributions presented in this study are included in the article/Supplementary Material. Further inquiries can be directed to the corresponding author.

## Supplementary Materials

The following supporting information can be downloaded at: https://www.mdpi.com/article/10.3390/nu17213386/s1, Table S1: The sequences of siRNA and plasmids; Table S2: Primer sequences; Table S3: Primary and secondary antibodies and dilution ratio.

## Abbreviations

The following abbreviations are used in this manuscript:

BC	Breast Cancer
MEL	Melatonin
BMAL1	Brain and Muscle ARNT-Like 1
CLOCK	Circadian Locomotor Output Cycles Kaput
PER	Period
CRY	Cryptochrome
RORA	RAR-Related Orphan Receptor A
NR1D1/2	Nuclear Receptor Subfamily 1 Group D Member 1/2
SIRT1	Sirtuin 1
GPAM	Glycerol-3-Phosphate Acyltransferase Mitochondrial
EZH2	Enhancer of Zeste Homolog 2
ALDH3A1	Aldehyde Dehydrogenase 3 Family Member A1
