# Supplementary material for: Natural Compound Melatonin Suppresses Breast Cancer Development by Regulating Circadian Rhythm
Source: Nutrients. 2025 Oct 28;17(21):3386. doi: 10.3390/nu17213386 (PMC12609774; doi:10.3390/nu17213386)
Supplement: Supplementary file 1 [file nutrients-17-03386-s001.zip › nutrients-3920937-supplementary.pdf]

Supplementary Material

Table S1. The sequences of siRNA and plasmids.

SiBMAL1	GCUCUUUCUUCUGUAGAAUTT(sense)
	AUUCUACAGAAGAAAGAGCTT(anti-sense)
SiNC	UUCUCCGAACGUGUCAC GUTT(sense)
	ACGUGACACGUUCGGAGAATT(anti-sense)
pcDNA3.1(+)/BMAL<br>1	GGATCCATGGCAGACCAGAGAATGGACATTTCTTCAACCATCAGTGA<br>TTTCATGTCCCCGGGCCCCACCGACCTGCTTTCCAGCTCTCTTGGTAC<br>CAGTGGTGTGGATTGCAACCGCAAACGGAAAGGCAGCTCCACTGAC<br>TACCAAGAAAGCATGGACACAGACAAAGATGACCCTCATGGAAGGT<br>TAGAATATACAGAACACCAAGGAAGGATAAAAAATGCAAGGGAAGC<br>TCACAGTCAGATTGAAAAGCGGCGTCGGGATAAAATGAACAGTTTTA<br>TAGATGAATTGGCTTCTTTGGTACCAACATGCAACGCAATGTCCAGG<br>AAATTAGATAAACTTACTGTGCTAAGGATGGCTGTTCAGCACATGAA<br>AACATTAAGAGGTGCCACCAATCCATACACAGAAGCAAACTACAAA<br>CCAACTTTTCTATCAGACGATGAATTGAAACACCTCATTCTCAGGGCA<br>GCAGATGGATTTTTGTTTGTCGTAGGATGTGACCGAGGGAAGATACTC<br>TTTGTCTCAGAGTCTGTCTTCAAGATCCTCAACTACAGCCAGAATGAT<br>CTGATTGGTCAGAGTTTGTTTGACTACCTGCATCCTAAAGATATTGCC<br>AAAGTCAAGGAGCAGCTCTCCTCCTCTGACACCGCACCCCGGGAGC<br>GGCTCATAGATGCAAAAACTGGACTTCCAGTTAAAACAGATATAACC<br>CCTGGGCCATCTCGATTATGTTCTGGAGCACGACGTTCTTTCTTCTGTA<br>GGATGAAGTGTAACAGGCCTTCAGTAAAGGTTGAAGACAAGGACTT<br>CCCCTCTACCTGCTCAAAGAAAAAAGCAGATCGAAAAAGCTTCTGC<br>ACAATCCACAGCACAGGCTATTTGAAAAGCTGGCCACCCACAAAGA<br>TGGGGCTGGATGAAGACAACGAACCAGACAATGAGGGGTGTAACCT<br>CAGCTGCCTCGTCGCAATTGGACGACTGCATTCTCATGTAGTTCCACA<br>ACCAGTGAACGGGGAAATCAGGGTGAAATCTATGGAATATGTTTCTC<br>GGCACGCGATAGATGGAAAGTTTGTTTTTGTAGACCAGAGGGCAACA<br>GCTATTTTGGCATATTTACCACAAGAACTTCTAGGCACATCGTGTTATG<br>AATATTTTCACCAAGATGACATAGGACATCTTGCAGAATGTCATAGGC<br>AAGTTTTACAGACGAGAGAAAAAATTACAACTAATTGCTATAAATTT<br>AAAATCAAAGATGGTTCTTTTATCACACTACGGAGTCGATGGTTCAGT<br>TTCATGAACCCTTGGACCAAGGAAGTAGAATATATTGTCTCAACTAAC<br>ACTGTTGTTTTAGCCAACGTCCTGGAAGGCGGGGACCCAACCTTCCC<br>ACAGCTCACAGCATCCCCCCACAGCATGGACAGCATGCTGCCCTCTG<br>GAGAAGGTGGCCCAAAGAGGACCCACCCCACTGTTCCAGGGATTCC<br>AGGGGGAACCCGGGCTGGGGCAGGAAAAATAGGCCGAATGATTGCT<br>GAGGAAATCATGGAAATCCACAGGATAAGAGGGTCATCGCCTTCTAG<br>CTGTGGCTCCAGCCCATTGAACATCACGAGTACGCCTCCCCCTGATG<br>CCTCTTCTCCAGGAGGCAAGAAGATTTTAAATGGAGGGACTCCAGAC<br>ATTCCTTCCAGTGGCCTACTATCAGGCCAGGCTCAGGAGAACCCAGG

	TTATCCATATTCTGATAGTTCTTCTATTCTTGGTGAGAACCCCCACATAGGTATAGACATGATTGACAACGACCAAGGATCAAGTAGTCCCAGTAATGATGAGGCAGCAATGGCTGTCATCATGAGCCTCTTGGAAGCAGATGCTGGACTGGGTGGCCCTGTTGACTTTAGTGACTTGCCATGGCCGCTGTAAGAATTC

Table S2. Primer sequences.

Gene	Forward primer	Reverse primer
BMAL1	GGATGTGACCGAGGGAAGAT	CGTCGTGCTCCAGAACATAAT
GAPDH	GGAGCGAGATCCCTCCAAAAT	GGCTGTTGTCATACTTCTCATGG
LEP	TTGGCCCTATCTTTTCTATG	GCATACTGGTGAGGATCTGT
PTEN	TGAGTTCCCTCAGCCGTTACCT	GAGGTTTCCTCTGGTCCTGGTA
Chip-ALDH3A1(site 1)	GGCCCTCTACATGTTCTCCA	CAAGGTCACACAGCCAGAGA
Chip-ALDH3A1(site 2)	TGTCATCGTCCACATCACCT	AGGTGGTCCCTCCTGAATTT
Chip-ALDH3A1(site 3)	TGAGGGCCATAGGATTTGAG	TTTCCCTCTCCACCTTGTTG

Table S3. Primary and secondary antibodies and dilution ratio.

**Target**	**Company**	**Cat.No.**	**Dilution ratio**
BMAL1	Proteintech	14268-1-AP	1:2000
ALDH3A1	OriGene	TA501105	1:2000
PFKM	Proteintech	55028-1-AP	1: 1,000
HK	Proteintech	22029-1-AP	1: 5,000
β-actin	Proteintech	60008-1-Ig	1: 5,000
anti-mouse HRP secondary antibody	Immunoway	RS0001	1:3000
anti-rabbit HRP secondary antibody	Immunoway	RS0002	1:3000
